# A longitudinal study of the peripheral and central auditory pathways in individuals with acute lymphoid leukemia

**DOI:** 10.1016/j.clinsp.2023.100234

**Published:** 2023-06-24

**Authors:** Jéssica Sales Vosgrau, Liliane Aparecida Fagundes Silva, Vicente Odone Filho, Carla Gentile Matas

**Affiliations:** a Department of Physical Therapy, Speech-language Pathology and Audiology, and Occupational Therapy, Faculdade de Medicina (FMUSP), Universidade de São Paulo, São Paulo, SP, Brazil; b Department of Pediatrics, Faculdade de Medicina (FMUSP), Universidade de São Paulo, São Paulo, SP, Brazil

**Keywords:** Auditory evoked potentials, Auditory perception, Hearing, Leukemia-lymphoblastic precursor cell lymphoma, Pharmacological treatment

## Abstract

**Objective:**

To characterize the peripheral and central auditory pathways in individuals with Acute Lymphoid Leukemia (ALL) and compare assessment results before and during chemotherapy.

**Method:**

The study included 17 subjects with ALL, divided into two age groups: 3 to 6 (11 individuals) and 7 to 16 years old (6 individuals). Each subject was evaluated twice (before and 3 to 6 months after chemotherapy treatment) with the following procedures: medical history survey, otoscopy, Pure-Tone Threshold (PTA) and speech audiometry, acoustic immittance measures, Brainstem Auditory Evoked Potentials (BAEP) and Long-Latency Auditory Evoked Potentials (LLAEP).

**Results:**

PTA was normal. Tympanometry was abnormal in the second assessment in 2 individuals aged 3 to 6 years. One subject in each age group had absent ipsilateral acoustic reflexes. In high-frequency audiometry, 1 individual had abnormal results. BAEP was abnormal in 5 (first assessment) and 7 individuals (second assessment) aged 3 to 6 years and 2 (first assessment) and 1 individual (second assessment) aged 7 to 16 years. As for LLAEP, P1 latency was increased in 5 (first assessment) and 7 individuals (second assessment) aged 3 to 6 years.

**Conclusion:**

No hearing loss was identified in the behavioral audiological assessment. BAEP was more affected in the 3-to-6-year-old group, with greater impairment in the lower brainstem in the first and second assessments. In LLAEP, P1 was the most impaired component in children aged 3 to 6 years, and P2 and N2 were so for those 7 to 16 years old, especially in the second assessment.

## Highlights

• Acute lymphoid leukemia changes the central auditory pathway.
• Changes in the central auditory pathway in individuals with acute lymphoid leukemia are due to neurotoxicity.

## Introduction

Acute Lymphoid Leukemia (ALL) is the most common type of malignant neoplasm in children [1], with approximately 6000 cases diagnosed per year in the United States [2], and 75,000 new cases diagnosed worldwide. The highest incidence is among children aged 2 to 5 years, particularly white males 2, 3, 4, 5.

ALL originate from accumulated abnormal immature lymphoid cells in the bone marrow that can permeate the whole body and the central nervous system [2], hindering the proliferation of normal cells, and impairing the normal production of red blood cells, leukocytes, and platelets 5, 6, 7.

There are some risk factors for the development of ALL, such as prenatal exposure to X-Rays, postnatal exposure to high doses of radiation, previous treatment with chemotherapy, some genetic conditions (Down syndrome and neurofibromatosis), exposure to chemical products, drugs, immune factors, associated congenital factors, and predisposition to hematological diseases 8, 9, 10.

In general, the drugs used in treatment include prednisone, vincristine, L-asparaginase, daunorubicin, MADIT, cyclophosphamide, cytarabine, 6-Mercaptopurina, methotrexate, dexamethasone, doxorubicin, and tioguanine [2,3,6,7].

However, some of these drugs are ototoxic – i.e., they have toxic substances that can affect hearing and cause progressive lesions in cochlear sensory cells and destroy them 11, 12, 13.

Moreover, the literature points to some of these drugs as neurotoxic, damaging the central and/or peripheral nervous systems [7,13].

Auditory Evoked Potentials (AEP) assess the whole peripheral and central auditory systems [14]. AEP is an objective method that verifies the neuroelectric activity of the central auditory pathways in response to stimuli or acoustic events. They are analyzed regarding response latency and classified as short-, middle-, or long-latency potentials [15].

Brainstem AEP (BAEP) is a short-latency potential whose responses occur in the first 10 ms after the sound stimuli were presented [16]. It is a simple, objective, noninvasive method that assesses electrical activity from the auditory nerve to the upper brainstem [17].

Long-Latency AEP (LLAEP) generates a series of waves that occur 50 ms after the acoustic stimuli were presented, originating in afferent and efferent connections between the thalamus and prefrontal cortex. These connections are responsible for detecting, perceiving, discriminating, recognizing, and classifying auditory stimuli [17,18].

Given the few studies on the hearing of individuals with ALL submitted to chemotherapy, it is greatly important to investigate their auditory pathways from the middle ear to the auditory cortex to help early identify changes in their peripheral and central auditory pathways that might be related to ALL drug treatment.

This study hypothesizes that individuals with ALL submitted to chemotherapy have peripheral and central hearing impairments.

## Materials and methods

This is a cohort study of individuals with ALL submitted to chemotherapy, referred by the Institute for the Treatment of Child Cancer (ITACI, in Portuguese) and the present study followed the STROBE Statement guidelines.

This research, conducted at ITACI, was approved by the Ethics Committee for the Analysis of Research Projects (CAPPesq) of the Clinical Board of the Medical School Clinics Hospital at the University of São Paulo (FMUSP), under number 1.556.648.

The final sample comprised 17 subjects with ALL, divided into age groups – 3 to 6 (11 subjects) and 7 to 16 years old (six subjects).

Each subject was assessed at two different moments – first, before beginning the chemotherapy treatment, and then 3 to 6 months after the first assessment.

The following material and equipment were used in each assessment:

1 Protocol to collect the children's medical history, developed and used in the Department of Clinical Audiology of the Speech-Language-Hearing Program, at the Department of Physical, Speech-Language-Hearing, and Occupational Therapy at FMUSP.
2 Otoscope manufactured by Heine, model Mini Heine 2000, to inspect the external auditory meatus.
3 Middle-ear analyzer manufactured by Interacoustics, models AT235 and Zodiac 90, to take acoustic immittance measures.
4 Audiometer manufactured by Otometrics, model Itera II, and supra-aural earphones, model TDH-50, meeting ANSI S3.6–1989 and IEC-1988 standards. Sound booth complying with ANSI S3.1–1991 norms for levels of environmental noise in PTA, speech, and high-frequency audiometry.
5 Equipment manufactured by Intelligent Hearing System, model Smart EP, for electrophysiological hearing assessments with AEP, five copper surface electrodes, insert earphones model ER 3-A, and disposable test plugs.
6 Abrasive and electrolytic paste and micropore tape.

The following procedures were used:

Medical history survey to obtain their personal and otologic history.
Inspection of the external auditory meatus to rule out any outer ear impairment that might hinder the procedures.

PTA: Hearing thresholds were surveyed at 500, 1000, 2000, 4000 Hz, and, if possible, 8000 Hz. The normal criteria were defined as hearing thresholds equal to or lower than 15 dB for children under 7 years old [19] and mean hearing thresholds at 500, 1000, 2000, and 4000 Hz lower than 20 dB for those above 7 years old [20].

Speech audiometry: The Speech Recognition Threshold (SRT) and Speech Recognition Percentage Index (SRPI) were verified with word lists read aloud. The normal SRT criteria were responses equal to or below 15 dB above the mean hearing thresholds at 500, 1000, and 2000 Hz in PTA [21].

High-frequency audiometry: Hearing thresholds surveyed at 9000, 10,000, 11,200, 12,500, 14,000, and 16,000 Hz, using Geyer's values as a reference [22].

Acoustic immittance measures: The tympanogram was characterized according to Jerger's criteria (1970) [23], and ipsilateral and contralateral acoustic reflexes were verified at 500 to 4000 Hz, classified as either present or absent.

BAEP: Responses were picked up with the active electrode (Fz) and ground electrode (Fpz) positioned on the forehead and the reference electrodes, on the left (M1) and right mastoids (M2). BAEP was surveyed with rarefaction polarity click stimuli, presented monaurally at 80 dBnHL, at a presentation rate of 19.0 clicks per second, lasting 0.1 milliseconds, totaling 2000 stimuli. Tracing reproducibility was verified, confirming the existence of responses. Waves I, III, and V and interpeak intervals I‒III, III‒V, and I‒V were identified and analyzed in the tracing. The results were classified as either normal or abnormal, as proposed in the Biologic-Evoked Potential User Manual (1993) [24].

Abnormal results were described regarding the type of change, as follows: changes in the Lower Brainstem (LBS), with increased latency values in waves III and V and/or interpeak intervals I‒III and I‒V; changes in the Upper Brainstem (UBS), when the latency values of wave V and/or interpeak intervals I‒V and III‒V were increased while absolute latencies in waves I and III were normal; changes in the lower and upper brainstem (LBS + UBS), when LBS and UBS were found simultaneously in the same person [25].

LLAEP: To pick up LLAEP components P1, N1, P2, N2, and P3, the active electrode was positioned on the vertex (Cz), the reference electrodes were positioned on the right and left mastoids (M2 and M1), and the ground electrode was positioned on the forehead (Fpz).

Patients under 7 years old (i.e., 3 to 6 years old) watched a mute video during the procedure, and the tracing was obtained with tone-burst stimuli at 1000 Hz lasting 100 ms at a presentation rate of 1.1 stimuli per second, presented monaurally at 70 dBnHL with insert earphones ER-3A, totaling 512 stimuli in an 800 ms analysis window. Two tracings were obtained for each ear to ensure wave reproducibility.

Patients above 7 years old (i.e., 7 to 16 years old) performed a cognitive task while tone-burst stimuli were presented monaurally at 75 dBnHL, at a presentation rate of 1.1 stimuli per second, totaling 300 stimuli. Frequent stimuli were presented at 100 Hz, and rare ones, at 1500 Hz; 15% of them were rare stimuli, and the patient was instructed to mentally count every time the rare stimuli appeared – which was the cognitive task.

After recording, P1 latency was analyzed in patients under 7 years old, and P1, N1, P2, N2, and P3 latencies were analyzed in those above 7 years old.

LLAEP was also classified as normal or abnormal, as follows: normal when P1, N1, P2, N2, and P3 latency values met McPherson's normal criteria (1996) [18] for children 5 to 12 years and above 12 years old; delayed when the latencies in these components were higher than the normal values; and absent when the component was not found.

The data were tabulated and submitted to quantitative and qualitative statistical analyses. The quantitative data analysis described the mean, median, standard deviation, and minimum and maximum values of each assessment result. Procedure results were compared between the right and left ears with the ANOVA test. The qualitative data analysis described the proportion of abnormal results and the types of changes, following the abovementioned assessment criteria. The Pearson Chi-Square or Fisher exact test was used to verify the association between two categorical variables, such as the presence or absence of responses or changes (normal/abnormal) in the comparison between right and left ears. In all analyses, the significance level was set at p-value ≤ 0.05 (5%) [26,27].

## Results

The sample was characterized by the age and age group of individuals with ALL.

### Characterization of the sample

Table 1.

**Table 1:** Characterization of the sample (individuals with ALL submitted to chemotherapy) regarding sex, age group, and cerebrospinal fluid examination result (search for neoplastic cells).

	Mean	Standard deviation	Minimum	Maximum
**Age (years)**	7.2	4.36	3	16
	**Number of participants (N)**		**Percentage (%)**	
**Sex**				
Males	7		41.17%	
Females	10		58.82%	
**Cerebrospinal fluid examination result (search for neoplastic cells)**	Positive (N) 0		Negative (N) 17	

### PTA and speech audiometry

No indication of abnormal results was found in PTA in either the first or second assessment. As for speech audiometry, all SRT and SRPI results were compatible with the hearing thresholds found in PTA.

### Acoustic immittance measures

In tympanometry, two individuals in the 3-to-6-year-old age group had abnormal results in the second assessment (18.18%), characterized by a type C tympanogram.

Acoustic reflexes were absent in only one individual (in the first assessment) in each age group (3 to 6 and 7 to 16 years old).

### High-frequency audiometry

High-frequency audiometry was performed in individuals above 7 years old. Only one of them had an abnormal result, which occurred in both the first and second assessments.

### BAEP

Individuals with ALL were grouped per age, considering that the normal standard is the same for individuals above 3 years old.

No statistically significant differences were found between the right and left ears regarding quantitative analysis results of BAEP absolute and interpeak latencies in either of the two assessments (Tables 2 and 3).

**Table 2:** Descriptive analysis of absolute latency values (in ms) of waves I, III, V and interpeak intervals (in ms) I‒III, III‒V, I‒V of BAEP in the right and left ears of individuals with ALL (n=17), in the first assessment.

	Ear	N	Mean	Standard deviation	Minimum	Median	Maximum	p-value^a^
**Wave I**	RE	17	1.57	0.13	1.25	1.6	1.8	1.000
	LE	17	1.57	0.13	1.25	1.65	1.75	
**Wave III**	RE	17	3.79	0.13	3.5	3.77	4.05	0.694
	LE	17	3.80	0.12	3.63	3.77	4.1	
**Wave V**	RE	17	5.69	0.12	5.45	5.7	5.9	0.685
	LE	17	5.68	0.13	5.4	5.7	5.9	
**Interpeak interval I‒III**	RE	17	2.21	0.18	1.9	2.2	2.7	0.694
	LE	17	2.23	0.15	2	2.2	2.6	
**Interpeak interval III‒V**	RE	17	1.9	0.11	1.75	1.85	2.1	0.481
	LE	17	1.87	0.11	1.7	1.85	2.15	
**Interpeak interval I‒V**	RE	17	4.11	0.15	3.85	4.15	4.45	0.844
	LE	17	4.11	0.16	3.75	4.1	4.55	

RE, Right Ear; LE, Left Ear; N, Sample Number.

*p-value with a statistically significant difference.

a p-value obtained with the ANOVA test.

**Table 3:** Descriptive analysis of absolute latency values (in ms) of waves I, III, V and interpeak intervals (in ms) I‒III, III‒V, I‒V of BAEP in the right and left ears of individuals with ALL (n=17), in the second assessment.

	Ear	N	Mean	Standard deviation	Minimum	Median	Maximum	p-value^a^
**Wave I**	RE	17	1.57	0.15	1.3	1.57	1.88	0.204
	LE	17	1.60	0.14	1.35	1.65	1.88	
**Wave III**	RE	17	3.77	0.18	3.3	3.75	4.05	0.206
	LE	17	3.83	0.17	3.5	3.85	4.22	
**Wave V**	RE	17	5.65	0.15	5.45	5.63	5.92	1.000
	LE	17	5.65	0.14	5.45	5.6	5.85	
**Interpeak interval I‒III**	RE	17	2.21	0.19	1.85	2.25	2.48	0.825
	LE	17	2.21	0.16	1.93	2.25	2.45	
**Interpeak interval III‒V**	RE	17	1.86	0.11	1.72	1.85	2.2	0.291
	LE	17	1.83	0.12	1.63	1.82	2.13	
**Interpeak interval I‒V**	RE	17	4.08	0.15	3.84	4.07	4.35	0.297
	LE	17	4.05	0.15	3.75	4.05	4.4	

RE, Right Ear, LE, Left Ear, N, Sample Number.

*p-value with a statistically significant difference.

a p-value obtained with the ANOVA test.

Likewise, no statistically significant differences were found in qualitative BAEP analysis results (normal and abnormal) between the first and second assessments in either age group (3 to 6 and 7 to 16 years old) (Tables 4 and 5). Nonetheless, abnormal results predominated in the second assessment among those 3 to 6 years old in contrast with the first assessment (Table 4).

**Table 4:** Comparative analysis of BAEP results (normal or abnormal) in individuals aged 3 to 6 years with ALL, between the first and second assessments.

		1st assessment		2nd assessment		p-value
		N	%	N	%	
**BAEP**	Normal	6	54.55	4	36.36	0.3918
	Abnormal	5	45.45	7	63.64	

N, Sample Number; %, Percentage; p-value obtained with Chi-Square test.

**Table 5:** Comparative analysis of BAEP results (normal or abnormal) in individuals aged 7 to 16 years with ALL, between the first and second assessments.

		1st Assessment		2nd Assessment		p-value
		N	%	N	%	
**BAEP**	Normal	4	66.67	5	83.33	>0.9999999
	Abnormal	2	33.33	1	16.67	

N, Sample Number; %, Percentage, p-value obtained with Fisher exact test.

### LLAEP

No statistically significant differences were found in the qualitative LLAEP analysis results (normal and abnormal) between the first and second assessments in either age group (3 to 6 and 7 to 16 years old) (Tables 6 and 7). However, abnormal P1 results predominated in the second assessment among those 3 to 6 years old (Table 6).

**Table 6:** Comparative analysis of LLAEP results (normal or abnormal) in individuals aged 3 to 6 years with ALL, between the first and second assessments.

		1st assessment		2nd assessment		p-value
		N	%	N	%	
**LLAEP Component P1**	Normal	6	54.55	3	30	0.4899
	Abnormal	5	45.45	7	70	

N, Sample Number; %, Percentage; p-value obtained with Chi-Square test.

**Table 7:** Comparative analysis of LLAEP results (normal or abnormal) in individuals aged 7 to 16 years with ALL, between the first and second assessments.

	Result	1st assessment (N)	2nd assessment (N)	p-value
**P1**	Normal	5	5	>0.9999999
	Abnormal	0	0	
**N1**	Normal	5	5	>0.9999999
	Abnormal	0	0	
**P2**	Normal	5	4	>0.9999999
	Abnormal	0	1	
**N2**	Normal	3	3	>0.9999999
	Abnormal	2	2	
**P3**	Normal	5	5	>0.9999999
	Abnormal	0	0	

N, Sample Number; p-value obtained with Fisher exact test.

Annex I show the patients’ evolution from the first to the second assessment.

**Annex I:** Chart 1 Summary of the first and second assessment results of subjects with ALL per age group.

Individuals aged 3 to 6 years														
Subject	PTA		Speech Audiometry		Tympanometry Curve		Acoustic Reflex		BAEP Threshold		BAEP Latency		LLAEP Latency	
	1st assessment	2nd assessment	1st assessment	2nd assessment	1st assessment	2nd assessment	1st assessment	2nd assessment	1st assessment	2nd assessment	1st assessment	2nd assessment	1st assessment	2nd assessment
1	N	N	N	N	N	N	N	N	NE	NE	A	A	N	N
2	N	N	N	N	N	N	N	N	NE	NE	N	A	A	A
3	N	N	N	N	N	N	N	N	N	N	N	N	N	N
4	N	N	N	N	N	N	N	N	N	N	N	N	N	N
5	N	N	N	N	N	N	N	N	N	N	A	A	A	A
6	N	N	N	N	N	N	A	N	N	N	N	A	A	A
7	N	N	N	N	N	N	N	N	NE	NE	N	N	A	N
8	N	N	N	N	N	N	N	N	NE	N	A	A	A	A
9	N	N	N	N	N	A	N	A	NE	NE	A	N	N	A
10	N	N	N	N	N	A	N	A	NE	NE	A	A	N	A
11	N	N	N	N	N	N	N	N	N	NE	N	A	N	A
**Individuals aged 7 to 16 years**														
**Subject**	**PTA**		**Speech Audiometry**		**Tympanometry Curve**		**Acoustic Reflex**		**HFA**		**BAEP Latency**		**LLAEP Latency**	
	**1st assessment**	**2nd assessment**	**1st assessment**	**2nd assessment**	**1st assessment**	**2nd assessment**	**1st assessment**	**2nd assessment**	**1st assessment**	**2nd assessment**	**1st assessment**	**2nd assessment**	**1st assessment**	**2nd assessment**
1	N	N	N	N	N	N	N	N	NE	NE	A	A	N	N
2	N	N	N	N	N	N	N	N	NE	NE	N	A	A	A
3	N	N	N	N	N	N	N	N	N	N	N	N	N	N
4	N	N	N	N	N	N	N	N	N	N	N	N	N	N
5	N	N	N	N	N	N	N	N	N	N	A	A	A	A
6	N	N	N	N	N	N	A	N	N	N	N	A	A	A

PTA, Pure-Tone Audiometry; BAEP, Brainstem Auditory Evoked Potentials; LLAEP, Long latency Auditory Evoked Potentials; HFA, High-Frequency Audiometry; N, Normal; A, Abnormal; NE, Not Evaluated.

## Discussion

All individuals in this study had normal results in the investigation of their peripheral auditory pathways with PTA and speech audiometry in both the first and second assessments.

Electrophysiological thresholds were surveyed with BAEP in five individuals who were not apt to undergo PTA due to fatigue, tiredness, or excessive sleepiness. They were normally brought straight from the general ward and were unable to cooperate with audiometry for a reliable result. Their electrophysiological threshold results were normal – i.e., at 20 dBnHL.

These results can be explained by the fact that ALL patients do not take cisplatin or carboplatin. As indicated in the specialized literature, chemotherapy drugs include prednisone, vincristine, L-asparaginase, daunorubicin, cyclophosphamide, cytarabine, 6-Mercaptopurine, methotrexate, dexamethasone, doxorubicin, etc [3,6,7].

Tympanometry was performed in all 17 patients included in this study. Two individuals 3 to 6 years old had abnormal results in the second assessment (18.18%), with type C tympanograms.

According to the literature, 22.3% of individuals submitted to chemotherapy complain of otitis, and 13.8% complain of upper airway infection [28].

All individuals 7 to 16 years old had normal results in both the first and second assessments.

Only one individual in each age group (3 to 6 and 7 to 16 years old) had absent acoustic reflexes, which occurred in the first assessment.

High-frequency audiometry was conducted in individuals above 7 years old, and only one of them had abnormal results, which occurred in both the first and second assessments. This subject's threshold was above the expected at 16 kHz in the right ear in the first assessment, and again at 16 kHz in both ears in the second assessment. Geyer's values (2015) [22] were used as a reference – i.e., thresholds up to 25 dBHL, according to previous studies in normal hearing individuals [29,30].

Patients submitted to ototoxic drugs during chemotherapy are known to be more likely to have high-frequency hearing loss, as they cause changes in the basal portion of the cochlea. It can also progress to the apical portion of the cochlea 11, 12, 13, as observed in the study by Korinthenberg and Igel (1990) [31], in which five out of the 26 patients submitted to chemotherapy had high-frequency hearing loss.

Specific mean latency values of waves I, III, and V and interpeak intervals I‒III, III‒V, and I‒V in the right and left ears in the first assessment are shown in Table 2, while those obtained in the second assessment are shown in Table 3. No statistically significant differences were found between the right and left ears in the two assessments.

The comparison of normal and abnormal BAEP results between the age groups (3 to 6 years and 7 to 16 years) (respectively, Tables 4 and 5) shows that in the first assessment, those 3 to 6 years old had a greater percentage of abnormal results (45.45%) than those 7 to 16 years old (33.33%). Such a difference was further evident in the second assessment – i.e., those 3 to 6 years old had an even greater percentage of abnormal results (63.64%) than those 7 to 16 years old (16.67%).

Regarding the types of changes found in the first assessment, three of the five individuals aged 3 to 6 years with abnormal results were classified with LBS changes (60%), while two of them had UBS changes (40%). In the second assessment, five of the seven individuals aged 3 to 6 years with abnormal results were classified with LBS changes (71.43%), while two of them had UBS changes (28.57%).

Few studies in the literature describe the analysis of auditory pathway integrity in the brainstem of individuals with ALL submitted to chemotherapy. The study by Kroczka et al. (2006) [32] assessed children with ALL after finishing chemotherapy, likewise using BAEP, and 22.4% of the patients had some type of change: one patient had increased latency in wave V and interpeak intervals I‒III and III‒V, another one was detected with increased interpeak intervals I‒III and I‒V, while another two were detected only with increased interpeak interval I‒III.

The results of the present study also agree with those obtained in the study by Leite et al. (2020) [33], who investigated auditory pathways in the brainstem of children with ALL submitted to chemotherapy (administered intravenously and intrathecally). They observed that 35.71% of the 14 children with normal hearing thresholds had abnormal BAEP results, with a predominance of impaired auditory pathways in the lower brainstem.

LLAEP was assessed in 17 individuals. In those aged 3 to 6 years, only P1 was researched, while P1, N1, P2, N2, and P3 were researched in those aged 7 to 16 years.

The number of individuals 3 to 6 years old with abnormal P1 results (increased latency) was greater in the second (70%) than in the first assessment, though with no statistically significant difference between the assessments.

More components (P1, N1, P2, N2, and P3) were abnormal in the second than in the first assessment in individuals aged 7 to 16 years, though with no statistically significant differences between the assessments. Moreover, all LLAEP component results were normal in the first assessment.

P1 was the most impaired LLAEP component in those aged 3 to 6 years in this study, while P2 and N2 were the most impaired ones in those aged 7 to 16 years. This cortical impairment is believed to be due to the neurotoxicity of the chemotherapy drugs, as they do not distinguish normal from cancer cells [34], impairing the acoustic stimulus processing speed, observed in delayed P2 and N2 latencies.

Considering that N2 is an endogenous potential influenced by intrinsic events (such as perception and cognition) [18], N2 findings may suggest that changes in perception and cognition are already taking place. They are first seen in N2 and may in the future be seen in P3, explaining why N2 is the LLAEP component with the highest percentage of abnormal results in the research.

Ototoxic and neurotoxic chemotherapy effects can occur through either the hematogenic (intravenous) route (which affects the cochlea and then the nervous transduction) or the intrathecal route (which directly affects the nerve).

Korinthenberg and Igel (1990) [31] emphasized that central auditory changes in patients with ALL may be caused by both disease progress and chemotherapy neurotoxic effects. All individuals in the present research had their cerebrospinal fluid examined soon before the second assessment, and all results were negative for neoplastic cells, indicating that the disease had not infiltrated into the central nervous system.

Thus, these results restate the hypothesis that changes observed in AEP are due to the neurotoxicity of certain drugs, rather than disease progression. This agrees with data obtained in the study by Leite et al. (2020) [33], which investigated auditory pathways in the brainstem of children with ALL submitted to chemotherapy (intravenous and intrathecal routes) and observed that 80% of the children with BAEP changes had taken methotrexate intrathecally less than 30 days before, and 40% had the highest cumulative doses of endovenous methotrexate.

These findings emphasize the importance of using AEP to assess individuals with ALL submitted to chemotherapy, given the neurotoxicity and ototoxicity of chemotherapy drugs.

## Conclusion

In conclusion, the longitudinal study of the peripheral and central auditory pathways of individuals with ALL shows that:

• Children 3 to 6 years old with ALL predominantly have:

Normal hearing in PTA.
Normal tympanometry and acoustic reflex results.
BAEP changes in the first and second assessments, with a predominance of changes in the auditory pathway in the lower brainstem, and more abnormal results in the second assessment.
Abnormal P1 in the first and second LLAEP assessments, characterized by increased latency and more abnormal results in the second assessment.

• Children 7 to 16 years old with ALL predominantly have:

Normal hearing in PTA.
Normal tympanometry and acoustic reflex results.
Normal high-frequency audiometry results.

Predominantly normal BAEP in the first and second assessments; changes were observed in the auditory pathway in the lower and upper brainstem.

In LLAEP, changes in N2 in the first assessment and P2 and N2 in the second assessment, are characterized by increased latency.

## Authors’ contributions

JSV: literature, data acquisition, and analysis, manuscript preparation and editing, review.

LAFS: preparation and editing, and final review.

VOF: concepts, design, literature, manuscript preparation and editing, final review.

CGM: concepts, design, literature, data analysis, statistical analysis, manuscript preparation and editing, and final review.

## Declaration of Competing Interest

The authors declare no conflicts of interest.
